# The Unfolded Protein Response: A Novel Therapeutic Target in Acute Leukemias

**DOI:** 10.3390/cancers12020333

**Published:** 2020-02-01

**Authors:** Alberto M. Martelli, Francesca Paganelli, Francesca Chiarini, Camilla Evangelisti, James A. McCubrey

**Affiliations:** 1 Department of Biomedical and Neuromotor Sciences, University of Bologna, 40126 Bologna, Italy; francesca.paganell16@studio.unibo.it; 2 CNR Institute of Molecular Genetics, 40136 Bologna, Italy; francesca.chiarini@cnr.it (F.C.); camilla.evangelisti@cnr.it (C.E.); 3 IRCCS Istituto Ortopedico Rizzoli, 40136 Bologna, Italy; 4 Department of Microbiology & Immunology, Brody School of Medicine, East Carolina University, Greenville, NC 27834, USA; mccubreyj@ecu.edu

**Keywords:** survival, apoptosis, genetic anomalies, targeted therapy, prognosis

## Abstract

The unfolded protein response (UPR) is an evolutionarily conserved adaptive response triggered by the stress of the endoplasmic reticulum (ER) due, among other causes, to altered cell protein homeostasis (proteostasis). UPR is mediated by three main sensors, protein kinase RNA-like endoplasmic reticulum kinase (PERK), activating transcription factor 6α (ATF6α), and inositol-requiring enzyme-1α (IRE1α). Given that proteostasis is frequently disregulated in cancer, UPR is emerging as a critical signaling network in controlling the survival, selection, and adaptation of a variety of neoplasias, including breast cancer, prostate cancer, colorectal cancer, and glioblastoma. Indeed, cancer cells can escape from the apoptotic pathways elicited by ER stress by switching UPR into a prosurvival mechanism instead of cell death. Although most of the studies on UPR focused on solid tumors, this intricate network plays a critical role in hematological malignancies, and especially in multiple myeloma (MM), where treatment with proteasome inhibitors induce the accumulation of unfolded proteins that severely perturb proteostasis, thereby leading to ER stress, and, eventually, to apoptosis. However, UPR is emerging as a key player also in acute leukemias, where recent evidence points to the likelihood that targeting UPR-driven prosurvival pathways could represent a novel therapeutic strategy. In this review, we focus on the oncogene-specific regulation of individual UPR signaling arms, and we provide an updated outline of the genetic, biochemical, and preclinical therapeutic findings that support UPR as a relevant, novel target in acute leukemias.

## 1. Introduction

Acute leukemias encompass a heterogeneous variety of blood malignancies characterized by a rapid clonal proliferation of immature hematopoietic cells displaying different levels of differentiation. If left untreated, acute leukemias typically lead to patient death within a few weeks. Acute myelogenous leukemia (AML) affects ~4.3 adults per 100,000 per year in the U.S. Typically, AML is a disease of the elderly, with a median age at diagnosis of 68 years [1]. Despite recent advances in understanding the genetic landscape of AML and its impact on the biology/pathophysiology of the disease as well as the availability of novel targeted therapeutics [2], the management of AML patients remains a challenge, especially in older adults who are ineligible for intensive therapies. Indeed, the long-term survival rate at 5-years after diagnosis is 40–50% in younger adults and ≤10% in older adults (≥65 years) [3]. Acute lymphoblastic leukemia (ALL) comprises B-cell ALL (B-ALL) and T-cell ALL (T-ALL). Overall, ALL is the second most common acute leukemia in adults, with an incidence of over 6500 cases per year in the U.S. [4], while B-ALL is the most common cancer in pediatric and adolescent patients where it comprises 80–85% of ALL cases (≥3000 cases per year in the US) [5]. B-ALL is curable by polychemotherapy alone in ~ 85% of children and 20–40% of adults [6,7]. Moreover, treatment of relapsed/refractory B-ALL has been revolutionized in recent years by the approval of blinatumomab, inotuzumab-ozogamicin, and chimeric antigen receptor (CAR) T-cell immunotherapy [8]. However, these new therapeutic approaches are not free from adverse effects and pose a substantial economic burden [9,10]. As to T-ALL, the 5-year overall survival in children is only slightly worse than in B-ALL, whereas adult patients show a trend towards a better survival rate (48% vs. 42%) [11]. Nevertheless, refractory/relapsed T-ALL patients still have poor outcomes, as no new pharmacological agents were specifically approved for T-ALL treatment since nelarabine in 2005 [12], while CAR-T immunotherapy poses many as yet unresolved challenges for the cure of T-cell malignancies [13]. Overall, there is still a clear need for novel and less toxic targeted therapies for all types of acute leukemias. Here, we discuss our current knowledge of the unfolded protein response (UPR) in acute leukemias, in relationship with their genesis, growth, and development of drug-resistance. We also highlight possible therapeutic strategies for exploiting the UPR as a novel target to ameliorate the outcome of acute leukemia patients.

## 2. Endoplasmic Reticulum Stress and the UPR

Given their high proliferation rate, it is essential for cancer cells to have a well-functioning machinery for meeting an increased demand for protein synthesis. The endoplasmic reticulum (ER) is the organelle responsible for the synthesis, maturation, stabilization, and folding of proteins embedded in the plasma membrane or destined to secretion. It is also responsible for the quality control of protein folding. Moreover, ER is involved in lipid and steroid synthesis, as well as in Ca2^+^-dependent signaling [14]. Both correct protein processing and folding are crucial for maintaining homeostasis not only of healthy but also of neoplastic cells. ER stress is one of the main factors that cause disturbances in these processes. ER stress is due to a variety of physiological and pathological perturbations, that include increased protein synthesis, an altered ubiquitin-proteasome system, anomalies in the autophagic machinery, Ca2^+^ depletion, pH changes, nutrient deprivation (e.g., hypoglycemia), increased levels of reactive oxygen species (ROS), inflammation, changes in the activity of oncogenes/tumor suppressor genes, and hypoxia [15,16,17]. These events interfere with the ER homeostasis, thereby causing an accumulation of unfolded/misfolded proteins that lead to ER stress [18]. Induction of ER stress then impacts on a dynamic and extremely complex intracellular signaling network referred to as the unfolded protein response (UPR). UPR is critical for sustaining the physiological functions of specialized secretory cells (e.g., pancreatic β cells, salivary gland secretory cells, and plasma cells [19,20,21]) and for controlling the production of lipids and cholesterol in the liver [22]. Three ER-anchored transmembrane stress sensors, protein kinase RNA-like endoplasmic reticulum kinase (PERK), inositol-requiring enzyme-1α (IRE1α) and activating transcription factor 6 (ATF6) mediate the correct function of the UPR in a time- and stimulus-specific manner, by triggering multiple cellular responses [23]. Through UPR, cells can adapt to unfavorable conditions and survive, for example by activating autophagy [24]. However, when the ER stress is unmitigated, the UPR switches from an adaptive cytoprotective to a cytotoxic proapoptotic response, that can be initiated also by ER-residing caspase-12 [25]. A failure in the re-establishment of proteostasis via deregulation of the UPR has been closely linked with a variety of human disorders, including neurodegenerative (e.g., Parkinson’s disease), cardiovascular, metabolic (e.g., type 2 diabetes), and autoimmune diseases, as well as cancer [26]. In particular, cancer cells are able to adapt to a prolonged ER stress via upregulation of the prosurvival UPR signaling. Activation of the UPR plays an important role during oncogenic transformation, as UPR signaling molecules closely interact with oncogene and tumor suppressor networks to modulate their functions. As UPR can lead to drug-resistance and emergence of cancer cell clones less sensitive to chemotherapy [27], a better understanding of the ER stress/UPR signals has the potential to lead to the development of efficacious therapeutic strategies for eradication of neoplastic cells. Indeed, treatment with pharmacological agents impacting on ER stress/UPR has shown promising outcomes in preclinical models of cancer [28]. The intricate signaling pathways leading to activation of the UPR and its functional consequences in solid tumors have been the subject of many recent comprehensive reviews [29,30,31,32,33,34,35,36,37,38]. Therefore, they will be only briefly summarized here. 

## 3. UPR Signaling

Under unstressed conditions, PERK, ATF6α, and IRE1α are kept inactive through binding to a luminal stress sensor protein, belonging to the 70-kDa heat shock protein family, referred to as 78-kDa glucose-regulated protein (GRP78), also known as binding immunoglobulin protein (BiP) [39]. Upon different stimuli, GRP78 binds with a higher affinity to the exposed hydrophobic domains of unfolded or misfolded proteins within the ER, thereby dissociating from the three ER-stress sensors [40]. Consequently, IRE1α and PERK can oligomerize and undergo autotransphosphorylation [41], while ATF6α translocates to the Golgi [42]. The three main UPR sensors display both common and unique downstream targets that orchestrate the appropriate responses. 

### 3.1. PERK

**Figure 1:**
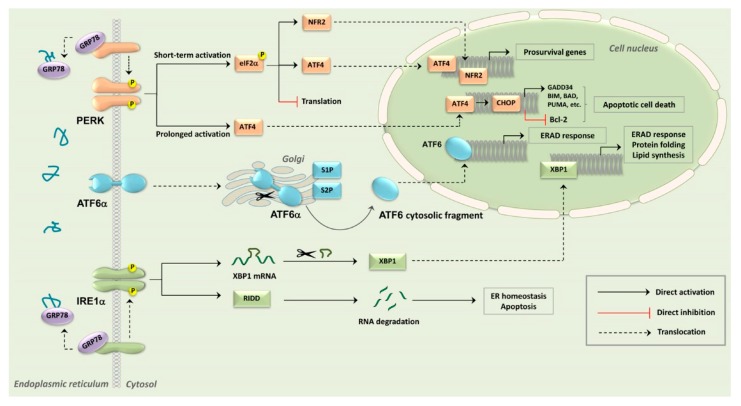
The three arms of the unfolded protein response (UPR). For the details see the text. Abbreviations used are: ATF4; Activating transcription factor 4; ATF6α: Activating transcription factor 6α; Bcl-2: B-cell lymphoma-2; BIM—Bcl-2 interacting mediator of cell death; CHOP—C/EBP-homologous protein; eIF2α—Eukaryotic translation initiator factor-2α; ER: endoplasmic reticulum; ERAD: ER-associated degradation; GADD34—Growth arrest and DNA damage-inducible protein 34; GRP78: 78-kDa glucose-regulated protein; IRE1α—Inositol-requiring enzyme-1α; NFR2: Nuclear factor (erythroid-derived 2)-like 2; PERK—Protein kinase RNA-like endoplasmic reticulum kinase; RIDD: Regulated IRE1α-dependent decay; S1P—Site-1 protease; S2P—Site-2 protease; XBP1—X-box binding protein 1.

PERK is a type I transmembrane kinase that, once activated by homodimerization and trans-autophosphorylation, phosphorylates eukaryotic translation initiator factor-2α (eIF2α) at Ser51 [43]. This phosphorylation attenuates global protein translation, thereby decreasing the overload of proteins entering the ER and reducing the protein-folding demand. However, phosphorylated eIF2α also allows the selective translation of the mRNA encoding activating transcription factor 4 (ATF4) (Figure 1). ATF4 then translocates to the nucleus, where it activates ER stress-response genes involved in protein folding, antioxidant response, hypoxia, and autophagy [44,45,46]. Moreover, active PERK phosphorylates nuclear factor (erythroid-derived 2)-like 2 (NFR2), a transcription factor belonging to basic leucine zipper domain (bZiP) proteins, that is involved in the control of antioxidant genes, stress response proteins, drug-metabolizing enzymes, and drug efflux transporters such as ATP-binding cassette (ABC) family members [47]. As a consequence of these events, short-term PERK activation results in prosurvival effects [42,48]. However, under severe and prolonged ER stress, prolonged ATF4 expression contributes to apoptotic cell death, as ATF4 upregulates C/EBP-homologous protein (CHOP) transcription factor levels (Figure 1). CHOP in turn increases the expression of several proapoptotic proteins. These include B-cell lymphoma-2 (Bcl-2) interacting mediator of cell death (BIM), Bcl-2 associated agonist of cell death (BAD), p53 upregulated modulator of apoptosis (PUMA), and phorbol-12-myristate-13-acetate-induced protein 1 (NOXA). In contrast, CHOP suppresses the transcription of prosurvival Bcl-2 [49]. These events result in the induction of mitochondria-dependent cell death. CHOP also positively impacts on the activity of c-Jun N-terminal kinase (JNK) that increases both death receptor and mitochondrial apoptosis [50]. Moreover, CHOP enhances the transcription of the *PPP1R15A* gene that encodes growth arrest and DNA damage-inducible protein 34 (GADD34), an eIF2α phosphatase [51]. Therefore, GADD34 dephosphorylates eIF2α and reverses translational inhibition, thereby switching-off one of the protective mechanisms of UPR. Indeed, the release of translational inhibition contributes to accumulation of unfolded proteins within the ER, while at the same time allows for the translation of mRNAs encoding proapoptotic proteins [52].

### 3.2. ATF6α

ATF exists as two isoforms, α and β. Of these isozymes, AT6α is the more relevant to the UPR [53]. ATF6α is a basic leucine zipper transcription factor that, upon ER stress induction, migrates from the ER to the Golgi for undergoing activation [54]. Two Golgi-residing peptidases, referred to as site-1 protease (S1P) and site-2 protease (S2P), sequentially cleave ATF6α on both sides of the Golgi membrane [55]. Following cleavage, the ATF6α p50 cytosolic fragment translocates to the nucleus where it activates transcriptional programs that promote ER stress adaptation, including protein folding and quality control as well as upregulation of various components of ER-associated degradation (ERAD) system (Figure 1). The ERAD system is essential for clearing unfolded/misfolded proteins from the ER [56].

### 3.3. IRE1α

The third and most evolutionary conserved signaling branch of the UPR is mediated by inositol-requiring enzyme-1 (IRE1), of which two isoforms exists, α and β. While IRE1α is ubiquitously expressed, IRE1β display a tissue-restricted expression. Although both isoforms are involved in ER stress, IRE1α is the better characterized of the two [57]. IRE1α contains both an endoribonuclease (RNase) domain on its cytosolic face and a kinase domain [58]. Upon ER stress induction, IRE1α trans-autophosphorylates and oligomerizes, thereby inducing a conformational change that activates the RNase domain (Figure 1). The best-known function of the RNase domain is a general downregulation of the ER load via unconventional splicing of the X-box binding protein 1 (XBP1) mRNA. The XBP1 mRNA unconventional splicing leads to the excision of a 26-nucleotide intron [59,60] ensuing in the generation of a stable and active transcription factor. The genes controlled by the spliced XBP1 variant encode a variety of proteins involved in the adaptive modulation of protein folding, secretion, and translocation to the ER, as well as in the ERAD and lipid synthesis [61,62,63]. However, XBP1 upregulates also the expression of several oncogenic factors, thereby promoting carcinogenesis, neoplastic cell survival, drug-resistance, and tumor progression [64]. Furthermore, IRE1α RNase activity is involved in controlling the RNA degradation pathway referred to as regulated IRE1α-dependent decay (RIDD) [65]. Although RIDD cleaves RNA at an XBP1-like consensus element (CUGCAG), its activity is divergent from canonical XBP1 cleavage [66]. RIDD has been associated with the degradation of many mRNA, rRNAs, and microRNAs (miRs), thereby either preserving ER homeostasis or inducing apoptosis [67]. For example, during RIDD, IRE1α cleaves and inactivates a selected subset of anti-CASP2 gene pre-miRs (miR-17, miR-34a, miR-96, and miR-125b). Consequently, there is an up-regulation of the *CASP2* gene and the initiation of the intrinsic apoptotic pathway [68]. IRE1α also acts as the driver of signals leading to increased expression of proapoptotic CHOP [67] and to the activation of the JNK pathway that facilitates cell death [69]. Therefore, IRE1α signaling plays a bifurcated role in determining cell fate during UPR.

## 4. UPR in Hematopoietic and Leukemic Stem Cells

Hematopoiesis, the hierarchical process of production of mature blood cells from the most immature cells, occurs in the bone marrow (BM), in an operationally defined compartment, known as the hematopoietic niche. Although earlier studies had suggested the existence of two different anatomical sites for the niche (periosteal and perivascular), preponderant evidence now favors the existence of only the latter [70]. The niche comprises hematopoietic stem cells (HSCs), hematopoietic progenitor cells (HPCs), and a variety of other components [vascular endothelial cells, C-X-C motif chemokine 12 (CXCL12)-abundant reticular cells, mesenchymal stromal cells (MSCs), adipocytes that form the BM microenvironment [38]. BM microenvironment cells nurture HSCs/HPCs via the production of a wide variety of factors (chemokines, cytokines), thereby providing the signals that lead to HSCs/HPCs survival, replication, and differentiation into all the mature blood cells [71]. Under steady-state conditions, HSCs are kept in a dormant state, so that they avoid metabolic and genotoxic stress due to mitosis [72]. Moreover, adult quiescent HSCs display a protein synthesis and folding capacity lower than those of their progeny, that probably reflects the dormancy of HSCs [73]. In contrast, when they are proliferating, HSCs need to increase protein translation to achieve expansion of the stem cell pool. However, due to their intrinsically poor protein folding activity, HSCs tend to accumulate unfolded/misfolded proteins that exceed the folding capacity and lead to the induction of the ER stress and proapoptotic UPR via the PERK-eIF2α branch. In contrast, HPCs exhibit an adaptive UPR response that leads to their survival [74]. Therefore, the higher vulnerability of HSCs to UPR has been proposed to represent a system for keeping a healthy pool of HSCs through the clearance of individual cells after ER stress, thereby preventing propagation of damaged HSCs and, ultimately, leukemogenesis [75]. However, a recent paper outlined that, when HSCs are stressed for a long period of time, they activate the IRE1α–XBP1 protective branch of UPR, that maintains HSC clonogenicity and reconstitution capacity [76]. It was also shown that dysregulated IRE1α signaling plays an important role in the leukemic transformation of HSCs into pre-leukemic stem cells (pre-LSCs) that subsequently evolve into LSCs [77]. LSCs fuel drug-resistance and disease relapses in acute leukemias [78]. LSCs actively compete with HSCs for the BM niche and are capable of remodeling the niche itself, which is transformed into a sanctuary site or leukemic niche. The leukemic niche hosts and protects LSCs [79]. Very recent findings have outlined that the presence of leukemic cells induces ER stress and UPR in both LSCs and healthy BM stromal cells of the niche. Extracellular vesicles were identified as the carrier of ER stress in vivo from leukemic cells to BM stromal cells, while the upregulation of UPR components (GRP78, XBP1, CHOP, phosphorylated IRE1α) drove the osteolineage differentiation of BM MSCs [80]. This observation is in agreement with the fact that UPR is a well-known stimulus for inducing osteogenic differentiation of MSCs [81]. It is noteworthy that a transmissible ER stress had been previously identified as a driver of chemoresistance in solid tumor models [82]. Therefore, a UPR-dependent remodeling of the BM niche into a leukemic niche could occur in acute leukemias and foster disease relapse.

## 5. UPR Involvement in AML

AML is a biologically complex, molecularly and clinically heterogeneous disorder [83]. It derives from myeloid-committed HPCs that originate a small population of self-renewing LSCs. In turn, LSCs give rise to a large population of immature leukemic blasts characterized by multiple epigenetic and genetic alterations [84,85,86]. These changes lead to aberrant activation of several signaling pathways that contribute to AML pathogenesis and progression [87,88,89,90]. In general, alterations in genes encoding epigenetic regulators are usually acquired earlier and are detectable in the founding pre-leukemic clone [84,85]. On the contrary, mutations involving nucleophosmin 1 (NPM1) or signaling molecules (e.g., FMS-like tyrosine kinase 3 (FLT3), or Rat sarcoma (Ras) gene family) are secondary events that occur later during leukemogenesis [85]. There are, however, exceptions to this rule. For instance, genetic alterations in transcription factor CCAAT/enhancer binding protein α (C/EBPα) drives the onset of AML both in mice and in humans [91], while the promyelocytic leukemia-retinoic acid receptor α (PML-RARα) fusion protein (i.e., the product of the balanced translocation t(15;17)) represents the main oncogenic driver of the acute promyelocytic leukemia (APL) AML subset [92]. 

**Figure 2:**
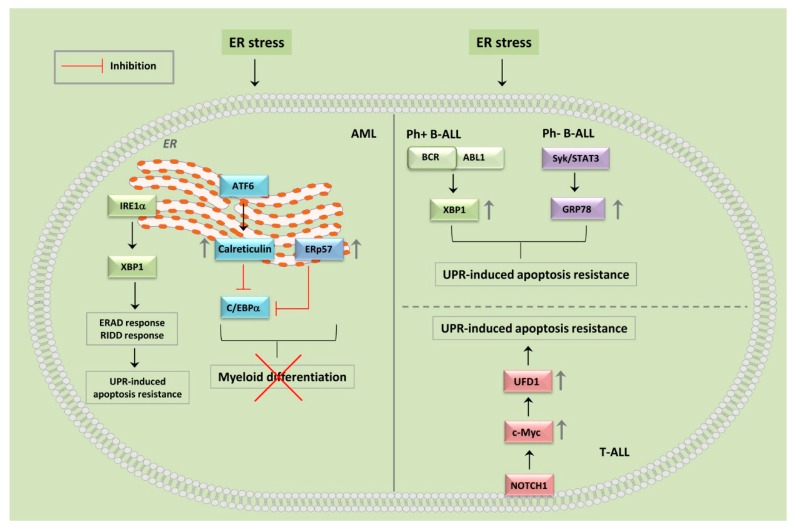
Involvement of unfolded protein response (UPR) in acute myelogenous leukemia (AML) and acute lymphoblastic leukemia (ALL). In AML cells (left panel), Inositol-requiring enzyme-1α (IRA1α)/X-box binding protein 1 (XBP1) enhanced signaling leads to ERAD—ER-associated degradation (ERAD) and IRE1α-dependent decay (RIDD) response, thereby causing resistance to apoptosis. Moreover, the activating transcription factor 6 (ATF6) branch of the UPR upregulates the expression of both Calreticulin and ER protein of 57 kDa (ERp57) that downregulate the expression of CCAAT/enhancer binding protein α (C/EBPα) leading to a myeloid differentiation block. In Philadelphia-negative (Ph^+^) B-ALL (upper right panel), upregulation of X-box binding protein 1 (XBP1) and 78-kDa glucose-regulated protein (GRP78) is controlled by Breakpoint cluster region-Abelson 1 (BCR-ABL1), whereas in Philadelphia-negative (Ph^−^) patients, it is downstream of the Spleen tyrosine kinase/Signal Transducer and Activator of Transcription 3 (Syk/STAT3) axis. In both cases, the outcome is the development of apoptosis resistance. In T-ALL with activated Neurogenic locus notch homolog protein 1 (NOTCH1) (lower right panel), there is an upregulation of C-Myc and ubiquitin fusion degradation 1 (UFD1), a component of the ERAD complex, which facilitates the elimination of misfolded/unfolded proteins from the endoplasmic reticulum (ER), thereby protecting T-ALL cells from apoptosis.

For the scopes of this review, it is important to emphasize that AML subsets are often characterized by the expression of fusion proteins prone to misfolding. Besides PML-RARα, mixed-lineage leukemia (MLL) fusion proteins are highly prevalent in pediatric AML patients and result from chromosomal translocations of the *MLL* gene (also referred to as MML-rearranged or MMLr) [93]. The MLL protein is a DNA methyltransferase (histone-lysine N-methyltransferase 2A) found with more than 60 fusion partners, thereby generating various acute leukemia subsets [94]. Hence, mutant and fusion proteins might enhance the sensitivity of AML cells to drug-induced disruption of proteostasis, as they are themselves a source of imbalanced proteostasis. It should also be considered that leukemic cells survive and proliferate in a hostile microenvironment characterized by low oxygen and pH, as well as by a limited nutrient availability [95]. Moreover, leukemic cells produce ROS at a high rate [96]. All these events lead to continuous ER stress with initiation of the UPR. The ability of leukemic cells to handle ER stress and a sustained UPR allow them to escape cell death and continue their growth. Regarding the UPR, the IRE1α-XBP1s branch was activated in ~18% of AML patients, as suggested by the increased expression levels of the spliced variant of XBP1, of GRP78, and of calreticulin (Figure 2) [97,98,99]. Interestingly, the outcome of patients with activated UPR was more favorable, as they displayed a lower relapse rate and a better overall/disease-free survival [97]. It should be considered that calreticulin is an ER chaperone protein involved in protein folding quality control and Ca2^+^ homeostasis [100,101]. When calreticulin was overexpressed in human U937 AML cells, it blocked the translation of C/EBPα, thereby negatively affecting myeloid differentiation [102]. In particular, in vitro studies demonstrated that calreticulin expression was upregulated via activation of the ATF6 pathway in AML cells, thereby ultimately suppressing translation of C/EBPα and contributing to the block in myeloid differentiation and cell-cycle deregulation which represent distinctive features of this leukemic phenotype. A similar role could be played by the ER protein of 57 kDa (ERp57), a thiol-disulfide oxidoreductase that resides in the ER lumen and interacts with calreticulin [103]. For instance, overexpression of ERp57 in AML cells blocked C/EBPα translation, but not its transcription, whereas abolishing ERp57 function restored C/EBPα protein levels. Importantly, induction of ER stress in human HL60 and U937 AML cells increased ERp57 expression, thereby decreasing C/EBPα protein levels (Figure 2) [104]. However, the exact mechanisms underlying C/EBPα downregulation still remain unexplained, as both calreticulin and ERp57 are not predicted to be present in the same cell compartment as C/EBPα mRNA. In any case, the observation that patients with activated UPR (and presumably lower C/EBPα levels) display a more favorable outcome is consistent with the notion that C/EBPα mutations and inactivation are associated with a better prognosis in AML [105]. c-Jun has been recently identified as a key transcriptional regulator of the UPR in AML. Indeed, upon ER stress induction, c-Jun is activated via mitogen-activated protein kinase kinase (MEK)/extracellular-regulated kinase (ERK) signaling and binds to the promoters of XBP1 and ATF4, thereby increasing their transcription and allowing leukemic cells to properly negotiate ER stress through cytoprotective UPR [106]. Of note, c-Jun/UPR signaling was activated by conventional chemotherapeutics (cytarabine, doxorubicine) used for AML patient treatment. This observation suggests that UPR activation might result in a lower efficacy of chemotherapy, as reported also by others [107]. Intriguingly, shRNA-mediated genetic ablation of XBP1 or ATF4 induced AML cell apoptosis and significantly extended disease latency in vivo in a murine model. Therefore, these findings tied the reduced AML cell survival observed when c-Jun activity was inhibited, to the loss of cytoprotective UPR signaling [106]. Moreover, the results identified the UPR as a promising potential therapeutic target in AML. However, the findings of Zhou et al. [106] are not easy to reconcile with those of others who reported a more favorable prognosis in patients with activated UPR [97], although the difference could simply reflect the opposite outcomes of the UPR in different AML settings. Indeed, c-Jun/UPR signaling upregulation correlated especially with AML cases the expressing MLL-AF9. i.e., the product of the 11q23 translocation t(9;11) [106].

### 5.1. UPR Involvement in APL

APL is a distinct subset of AML, which comprises about 15% of AML cases. PML-RARα alters the functions of both the transcription factor RARα and of the tumor suppressor PML, which is at the hub of the PML nuclear bodies (PML-NBs) [93]. PML-RARα disrupts the PML-NBs and acts as a transcriptional repressor through the block of RA-induced myeloid differentiation, thereby impeding the differentiation from the promyelocyte stage to granulocyte [108]. The PML-RARα fusion protein is not only a misfolded protein by itself, but also promotes aberrant folding of nuclear receptor corepressor 1 (NCOR1), a protein essential for the function of several tumor suppressors, including Max dimerization protein 1 (MAD1) [109]. 

**Figure 3:**
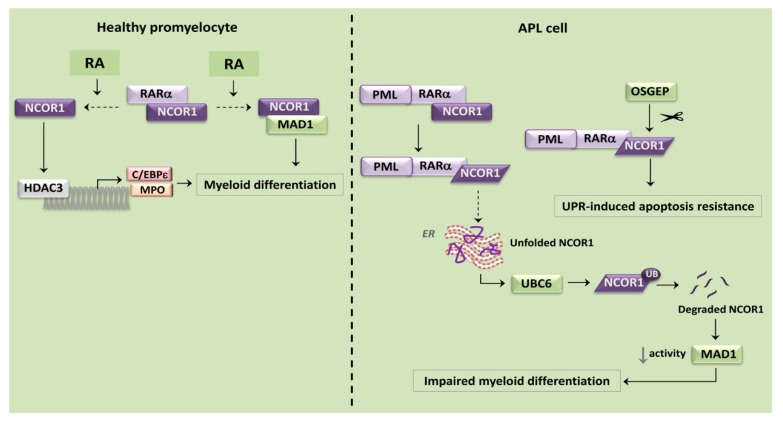
Involvement of unfolded protein response (UPR) in acute promyelocytic leukemia (APL). In healthy promyelocytes retinoic acid (RA) dissociates retinoic acid receptor α (RARα) from Nuclear receptor corepressor 1 (NCOR1). NCOR1 enhances myeloid differentiation through histone deacetylase 3-myeloperoxidase-CCAAT/enhancer binding protein ε (HDAC3-MPO-C/EBPε) signaling and association with the tumor suppressor Max dimerization protein 1 (MAD1) (left panel). In APL cells, promyelocytic leukemia-retinoic acid receptor α (PML-RARα) binds NCOR1 thereby inducing an abnormal conformation and insolubility of NCOR1. Therefore, aberrantly folded NCOR1 accumulates in the ER, eliciting ER stress and UPR. Furthermore, PML-RARα stimulates NCOR1 ubiquitinylation and degradation via ubiquitin-conjugating enzyme 6 (UBC6), an ER-associated degradation (ERAD) component involved in protein quality control. As a result of NCOR1 decreased expression, the tumor suppressive activity of MAD1 is downregulated and myeloid differentiation is impaired. Moreover, O-sialoglycoprotein endopeptidase (OSGEP) cleaves NCOR1, which results in resistance to UPR-induced apoptosis (right panel).

It should be considered that in healthy cells RARα forms heterodimers with NCOR1 that link chromatin-modifying enzymes (e.g., histone deacetylase 3) [110] with transcription factors specific for genes related to myeloid differentiation, including CCAAT/enhancer binding protein ε (C/EBPε) and myeloperoxidase (MPO) [111]. RARα releases NCOR1 in response to agonists such as RA, thereby enhancing myeloid differentiation (Figure 3) [112]. PML-RARα binding to NCOR1 leads to an abnormal protein conformation and insolubility of NCOR1 [109]. Hence, aberrantly folded NCOR1 accumulates in the ER, thereby eliciting ER stress and UPR [113]. Moreover, PML-RARα stimulates NCOR1 ubiquitinylation and degradation via the ubiquitin-conjugating enzyme 6 (UBC6), an ERAD component that is involved in protein quality control (Figure 3) [114]. As a consequence of NCOR1 decreased expression, the tumor suppressive activity of MAD1 is decreased and myeloid differentiation is impaired (Figure 3). Accordingly, forced expression of NCOR1 induces the differentiation of APL-derived NB4 human leukemia cells. This observation suggests that low NCOR1 expression levels may at least partly contribute to PML-RARα induced leukemogenesis [113]. Interestingly, APL cells are resistant to UPR-induced apoptosis. The resistance has been ascribed to the fact that these cells are characterized by the expression of O-sialoglycoprotein endopeptidase (OSGEP) that cleaves NCOR1 (Figure 3). OSGEP activity could be blocked by either the broad-spectrum protease inhibitor, 4-(2-aminoethyl)benzenesulfonyl fluoride hydrochloride (AEBSF) or through siRNA-mediated downregulation of OSGEP expression. AEBSF inhibited growth and promoted death of APL cells, most likely through a mechanism that involves AEBSF-induced accumulation of insoluble NCOR1 protein and triggers ER stress/cytotoxic UPR [115]. Overall, these findings highlight how misfolded NCOR1 is a potential conformation-based druggable target in APL cells. They also emphasize that the induction of a protease activity represents a component of cytoprotective UPR, which is exploited by APL cells for surviving the toxic insults due to the accumulation of misfolded proteins [116].

## 6. UPR Involvement in ALL

Similarly to AML, B-ALL presents a wide variety of genetic anomalies that include aneuploidy, fusion proteins (e.g., Ets-leukemia virus 6 - Runt-related transcription factor 1 (ETV6-RUNX1); breakpoint cluster region - Abelson1 (BCR-ABL1); rearrangements in the MLL gene; aberrations in key genes of B-cell development (e.g., paired box 5 (PAX5) and Ikaros family zinc finger protein 1 (IKZF1)) or cell cycle-related genes (CDKN2A); Neuroblastoma-Ras (N-Ras) point mutations (e.g., G12D). These molecular alterations define B-ALL progression and outcome [117,118]. The BCR-ABL1 fusion gene, that derives from the reciprocal translocation t(9;22)(q34;q11), originally defined a high-risk B-ALL subtype (Philadelphia-positive or Ph^+^ B-ALL) that correlated with a very poor outcome. The introduction of tyrosine kinase inhibitors (TKIs), such as imatinib, has improved the prognosis of this B-ALL subset. However, development of resistance to TKIs is still an unresolved issue [119,120]. At variance with B-ALL, no consensus genetic classification with either prognostic or therapeutic implications has not yet been reached for T-ALL. This could be related to the fact that most of T-ALL genetic anomalies are not predictive of patient outcome [118]. Nevertheless, in ≥60% of T-ALL patients, c-Myc is overexpressed downstream of activated Neurogenic locus notch homolog protein 1 (NOTCH1) mutations and plays a key role in disease induction and aggressiveness [121].

### 6.1. B-ALL

Regarding UPR, it should be highlighted that XBP1, IRE1α, and GRP78 are required for terminal differentiation of B-lymphocytes into plasma cells, hence they are expressed at high levels in plasma cell-derived MM, but have no clearly defined roles during the early stages of B-cell development [21]. However, their expression levels transiently peak at the pre-B-cell receptor checkpoint [122]. Several lines of evidence indicate that ER stress and UPR are somehow involved in both the pathophysiology and outcome of B-ALL. It was shown that the expression of XBP1, IRE1α, and GRP78 was upregulated in B-ALL patients, including BCR-ABL1 and MLLr B-ALL subsets, both at diagnosis and at relapse. Moreover, high levels of these proteins predicted a poor outcome [122,123]. XBP1 expression levels were under the control of BCR-ABL1 in Ph^+^ B-ALL subtype [122], whereas in Ph^-^ pediatric B-ALL patients GRP78 was downstream of the Spleen tyrosine kinase (Syk)/Signal Transducer and Activator of Transcription 3 (STAT3) axis [123] (Figure 2). Importantly, inducible deletion of XBP1 and GRP78 led to apoptosis in murine models of BCR-ABL1 and N-Ras-mutated B-ALL. Moreover, STF-083010, a small molecule inhibitor of the IRE1α RNase activity required for XBP1 mRNA splicing [124], was able to cause not only apoptosis, but also cell cycle arrest in B-ALL xenografts. This resulted into a prolonged survival of mice xenotransplanted with human B-ALL cells [122]. Overall, these findings revealed a unique vulnerability of B-ALL cells to ER stress/UPR and identified XBP1 as a novel therapeutic target [122]. 

### 6.2. T-ALL

Recent findings have highlighted c-Myc as a regulator of the UPR response in T-ALL cells via increased transcription of the ubiquitin fusion degradation 1 (UFD1) gene [125]. UFD1 is a component of the ERAD complex, which facilitates the elimination of misfolded/unfolded proteins from the ER (Figure 2). When UFD1 was inactivated in human T-ALL cells, ER stress was exacerbated and led to apoptosis via PERK-ATF4-CHOP signaling. Therefore, these findings identified c-Myc/UFD1 signaling as a critical regulator of the ER stress response in T-ALL with possible implications for novel targeted therapeutic strategies [125].

## 7. Therapeutic Targeting of UPR in Acute Leukemias

Since cancer cells frequently rely on UPR as an adaptive and defensive response, there is a growing interest in developing rationale treatments for aggravating ER stress to target the UPR, thereby inducing apoptosis. We shall now review the principal findings obtained in this field in preclinical models of both AML and ALL.

### 7.1. Therapeutic Strategies for AML

XBP1 promoter is hypomethylated in AML and this results in an expression level of the spliced form of XBP1 higher in AML cell lines and AML primary samples when compared to healthy CD34^+^ HSCs and HPCs [99]. Therefore, a panel of IRE1α RNase inhibitors [MKC-3946, 2-hydroxy-1-naphthaldehyde, STF-083010, and toyocamycin] was used for blocking XBP1 mRNA splicing. An interesting finding that emerged from this study is that IRE1α pharmacological inhibition significantly increased the levels of pre- and mature miR-34a, a well-established master regulator of tumor suppression [126]. Indeed, miR34a downregulate targets important for cancer cell proliferation and survival (cyclin D1, caspase-2, c-Myc, Bcl-2), while a downregulation of miR-34a leads to chemotherapy-resistance [126]. In AML cells, treatment with IRE1α inhibitors led to inhibition of cyclin D1, cyclin-dependent kinase 4 (CDK4), Bcl-2, c-Myc, and to an induction of p21^cip1^ and p27^kip1^, thereby causing both a G1 phase cell cycle arrest and a caspase-dependent apoptosis. These findings indicate the relevance of RIDD-dependent downregulation of miR-34a in the IRE1α-dependent prosurvival UPR in AML cells. They also support the concept that targeting IRE1α-driven prosurvival pathways may represent an alternative therapeutic approach for the treatment of some AML patients [99]. A very recent article has demonstrated that a combined treatment consisting of low concentrations of retinoic acid (RA), of the ER stressor tunicamycin, and of arsenic trioxide (ATO), led to ER and oxidative stresses as well as to UPR in AML cells expressing fusion proteins such as MLL-AF4, MLL-AF6, MLL-AF9, or FLT3-internal tandem duplication (ITD), alone or in combination. The treatment caused an accumulation of mutant FLT3 protein in the ER and decreased the colony-forming capacity of primary leukemic cells bearing the FLT3-ITD mutation without affecting healthy human HPCs isolated from the BM [127]. It is worth highlighting here that the FLT3-ITD fusion protein is detected in ~25–30% of AML cases and is a misfolded protein mostly retained in the ER. Indeed, ITD impairs full glycosylation and folding of FLT3 [128]. FLT3-ITD^+^ patients display a poor outcome after chemotherapy and after BM transplantation [129]. Several inhibitors of the overactive signaling unleashed by FLT3-ITD have been disclosed and tested in clinical trials. However, the available inhibitors, either used as monotherapy or combined with chemotherapy, yield incomplete and transient responses, with a rapid development of drug-resistance [130]. Importantly, the triple drug combination was effective in FLT3-ITD^+^ leukemic cells independently from the presence of other fusion proteins, thereby providing a proof of concept that such a treatment may be a promising therapeutic strategy for this high-risk AML subset. 

ER stress is very important in APL pathophysiology, as we have highlighted in Section 5.1. Therefore, it is not surprising that several groups have devised therapeutic strategies for targeting UPR in this AML subset. Historically, APL was considered one of the most aggressive subtypes of AML. However, therapeutic regimens based on RA combined either chemotherapy or with ATO, have resulted in drastically improved outcomes. In particular, the RA plus ATO combination provides a milder toxicity profile when compared with RA plus chemotherapy [131,132,133]. Therefore, the RA plus ATO treatment is a novel established therapeutic approach at least for non-high risk patients. However, RA and ATO are not devoid of adverse effects, which include the so-called RA-related differentiation syndrome, a potentially life-threatening condition [134]. Moreover, resistance to RA and ATO can develop, due to a number of reasons [92], including interactions between leukemic cells and the BM microenvironment [135]. It should be considered that PML-RARα degradation, driven by RA, restores both the functions of PML and the structure of nuclear bodies. Degradation of PML-RARα is dependent both on the ubiquitin–proteasome system and on autophagy, two processes that are highly interconnected with the functions of the ER. ATO synergizes with RA in PML-RARα degradation, moreover it induces APL cell death by increasing ROS levels and caspase activity [136]. It was observed that the natural diarylheptanoid, curcumin, exacerbated ER stress in APL cells and induced apoptosis, through the accumulation of aberrantly phosphorylated misfolded NCOR1 within the ER [137]. Curcumin promoted this effect by blocking the ERAD- and protease-mediated degradation of NCOR1 protein, which in turn led to PERK activation and upregulation of CHOP and GADD34, two key mediators of the cytotoxic UPR. Another proposed strategy to aggravate ER stress in APL cells is the use of the proteasome inhibitor bortezomib, one of the drugs that have revolutionized the treatment of MM [138]. Bortezomib increased the abundance of ubiquitininylated PML-RARα via an upregulation of ubiquitin-conjugating human enzyme 8 (UBCH8), the E2 ubiquitin-conjugating enzyme for PML-RARα [139]. This resulted in apoptosis in human NB4 APL cells. The efficacy of bortezomib in this preclinical setting is particularly interesting, as several clinical trials incorporating bortezomib in the therapeutic regimen have reported a range of good responses in AML patients [140]. However, the most promising application of ER stress modulators in APL is related to a possible combined treatment with RA or ATO, mostly with the aim to lower resistance to these drugs. Taking advantage of the ATO-resistant human APL cell line, NB4 EVAsR1, it was recently demonstrated that resistance could be effectively overcome by combining bortezomib with ATO [141]. Several mechanisms cooperated to achieve a synergy between bortezomib and ATO, including a downregulation of the nuclear factor-κB (NF-κB) pathway, an increased generation of ROS, an augmented autophagic degradation of PML-RARα and an upregulation of the proapoptotic UPR components, ATF4 and CHOP. The ATO-bortezomib combination was effective also in vivo in a xenografted APL murine model [141]. Importantly, these preclinical findings paved the way for testing the bortezomib/ATO combination therapy on a compassionate basis in five ATO-resistant, relapsed APL patients. Four patients were in continuous molecular remission for 40–60 months.

**Figure 4:**
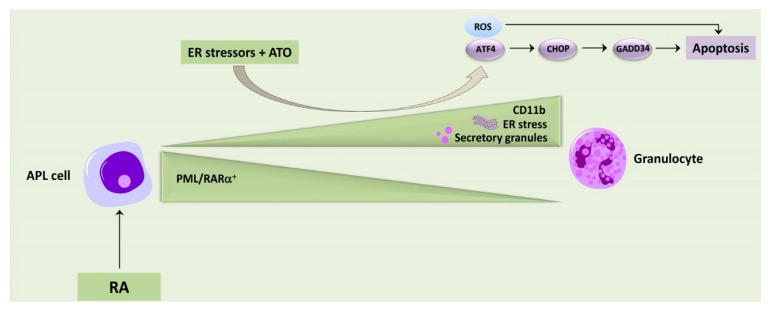
RA treatment drives differentiation of acute promyelocytic leukemia (APL) cells (as documented by the increased expression of the myeloid differentiation marker, CD11b, and the degradation of promyelocytic leukemia-retinoic acid receptor α (PML-RARα)) which is accompanied by enhanced endoplasmic reticulum (ER) stress due to the production of secretory granules. In presence of retinoic acid (RA), leukemic cells become more sensitive to low concentrations of ER stressors (e.g., tunicamycin) and to arsenic trioxide (ATO), that induce apoptosis via unfolded protein response (UPR) and reactive oxygen species (ROS). Other abbreviation used are: ATF4: Activating transcription factor 4; CHOP: C/EBP-homologous protein; GADD34: Growth arrest and DNA damage-inducible protein 34.

The combined treatment was well tolerated with no significant grade III/IV hematological adverse effects [141]. Following this preliminary favorable experience, a phase II clinical study for relapsed APL patients was started in 2013 (https://clinicaltrials.gov/NCT01950611). Although the results from this trial have not been published yet, we know from the article by Ganesan and coworkers [141] that all the 16 patients enrolled at the time of article submission were still in molecular remission. Again, except for one patient who developed grade III peripheral neuropathy (most likely due to bortezomib [142]), none of the other patients displayed any grade III/IV adverse effects. Therefore, the combination consisting of bortezomib with ATO demonstrated the potential to be efficacious in high-risk and relapsed/resistant APL patients. It should also be considered that RA upregulates ER stress while inducing the differentiation of APL cells towards mature granulocytes, that is characterized by the production of secretory granules and an increased demand for protein folding in the ER (Figure 4) [143]. Interestingly, it was recently reported that RA-induced differentiation of human APL cell lines and primary cells blasts led to an increase of their sensitivity to ER stress-inducing drugs (either tunicamycin or thapsigargin) at concentrations that were not cytotoxic in the absence of RA. Furthermore, low concentrations of ER stress-inducing drugs were sufficient to markedly increase ATO cytotoxicity also in RA-resistant APL cell lines [143]. Importantly, APL cells treated with RA or with RA/tunicamycin degraded PML-RARα to a similar extent, which implies that increased PML-RARα degradation could not explain the therapeutic benefits of tunicamycin. Indeed, it was shown that ER stress generated a more oxidative environment that enhanced ATO-dependent apoptosis. In this context, it was also demonstrated that the ATF4-CHOP-GADD34 axis played a decisive role in the induction of apoptosis in response to ER stress in APL cells (Figure 4) [143]. Therefore, these results confirm the relevance of ER stress-elicited signaling pathways as potential targets for the treatment of RA-resistant APL patients.

### 7.2. Therapeutic Strategies for B-ALL and T-ALL

Several lines of evidence advocate the use of ER stressors as potential drugs for the treatment of both B-ALL and T-ALL. Earlier studies focused on how the AMP-activated kinase (AMPK) activator 5-Aminoimidazole-4-carboxamide ribonucleotide (AICAR [144]) in combination with either methotrexate or 2-deoxy-D-glucose (2-DG, a sugar analog that inhibits both glycolysis and N-linked glycosylation [145]), induced a prolonged ER stress in B-ALL cells, thereby leading to proapoptotic UPR via IRE1α, GRP78, phosphorylated eIF2α, and CHOP [146]. Similar results were reported with the AMPK activator metformin that led to UPR-mediated apoptosis via upregulation of IRE1α and CHOP [138]. Interestingly, Ph^+^ B-ALL cells displayed the highest sensitivity to 2-DG and ER-stressors (i.e., either tunicamycin or thapsigargin), as these compounds translationally repressed prosurvival Myeloid cell leukemia 1 (Mcl-1) protein by activating the AMPK mechanistic target of rapamycin (mTOR) and UPR/PERK/eIF2α signaling pathways. The expression of Mcl-1 was further reduced when 2-DG and ER-stressing agents were combined with TKIs (imatinib, dasatinib, nilotinib) [147]. Similar findings were reported also in T-ALL settings, where methotrexate, AICAR or metformin in combination with 2-DG induced a sustained ER stress, thereby leading to UPR and apoptosis [146,148]. Interestingly, metformin induced ER stress also in a cohort of heavily pretreated pediatric ALL patients subjected to an induction chemotherapy consisting of vincristine, dexamethasone, PEG-asparaginase, and doxorubicin [149]. These findings further strengthen the contention that AMPK activation impacts on the UPR, leading to increased vulnerability to ER stress in ALL cells. Other strategies aimed to unleash proapoptotic UPR in ALL cells involved the use of Pevonedistat^®^ (Takeda Oncology, Cambridge, MA, USA), an inhibitor of neural precursor cell expressed, developmentally down-regulated 8 (NEDD8)-activating enzyme that targets E3 cullin-RING ligases-dependent proteasomal protein degradation [150]; of CX-4945, an inhibitor of casein kinase 2 (CK2) signaling [151], also in combination with bortezomib [152]; of the natural acyclic sesquiterpene alcohol, farnesol [153]; of sphingosine kinase inhibitors [154]; of a phosphine copper(I) complex [Cu(thp)4][PF6] [155]; of a combination of ATO and dasatinib that was effective in Ph^+^ B-ALL cells [156]; of epigallocatechin gallate, a green tea extract, that binds to the ATP-binding site of GRP78, thereby leading to a conformational conversion into its inactive oligomeric form [123]. All these studies demonstrated that ALL cells are prone to ER stress/UPR-mediated apoptosis via a variety of signaling pathways/mechanisms. Therefore, the use of ER stressors as potential drugs for the treatment of some refractory/relapsed forms of both B-ALL and T-ALL might be considered. Of note, a phase I clinical study involving the use of Pevonedistat^®^ in combination with chemotherapy has been started in 2019 for adolescent/young adults (16-39 years of age) with relapsed/refractory ALL (https://clinicaltrials.gov/NCT03349281). 

## 8. Conclusions

As we have summarized in this article, ER stress/UPR signaling is being increasingly recognized as a promising target for the development of new therapies for acute leukemias. Indeed, activation of this intricate network represents an important step during oncogenic transformation and influences several hallmarks of leukemic cells, including drug-resistance. Therefore, ER stress/UPR modulators seem to be promising drugs for a personalized therapy of patients in whom conventional chemotherapy or other more established targeted therapies (TKIs, RA, ATO) have failed. Several therapeutic strategies for targeting ER stress/UPR are now available [42], including PERK inhibitors (GSK2606414, GSK2656157), IRE1α inhibitors (MKC-3946, 8-formyl-7-hydroxy-4-methylcoumarin, STF-083010), and ATF6α modulators, such as Ceapins that specifically inhibit ATF6α signaling by blocking its processing and nuclear translocation [157]. However, the dual impact of the UPR on the cell fate strongly indicates that therapeutic targeting of this intricate network requires a complete understanding of the precise events dictating whether the UPR promotes cell death or survival [158]. Indeed, an activated UPR could be related to a better patient outcome in some AML settings, as we have outlined in Section 5. Obviously, in this case treatment with UPR inhibitors might have a detrimental effect on disease progression. Despite the relevance of ER stress/UPR in hematological disorders, at present there are only two on-going clinical studies in which modulation of the UPR was set as a therapeutic target in acute leukemia patients, i.e., the aforementioned NCT01950611 and NCT03349281. There are however, many completed or on-going studies in which bortezomib or metformin have been used for treating acute leukemia patients, either alone or in combination with other drugs (https://clinicaltrials.gov). Although targeting ER stress/UPR signaling has great potential for intervention in acute leukemias, further studies are warranted to answer several unresolved issues. Acute leukemias are highly heterogeneous diseases. Hence, an elucidation of how different oncogenes/tumor suppressors impact on ER stress/UPR is required. Moreover, the relevance of ER stress/UPR in LSCs is completely unknown and needs to be clarified, if we aim to the eradication of the disorder [159]. Furthermore, the length of drug exposure should be taken into consideration due to the dynamic and time-dependent cytotoxic or cytoprotective effects of the UPR [158]. A further layer of complexity is added by the interactions between leukemic cells and the BM microenvironment, especially in light of the transmissibility of ER stress and UPR between cells via extracellular vesicles, that can transport mRNAs, miRs, and misfolded proteins, thereby disrupting ER homeostasis of the recipient cells [67,160]. ER stress transmitted by leukemic cells via extracellular vesicles results in changes in the composition and function of BM microenvironmental cells, leading to the osteogenic differentiation of BM MSCs [80]. Therefore, therapeutics specifically targeting signaling pathways that control the interactions between leukemic BM niche osteoblasts/osteocytes and LSCs, might represent a strategy for lowering the drug-resistance of AML cells and eradicating the disease [161,162]. Last, but not the least, it will be important to develop treatments targeting leukemic cell intrinsic aberrations (both epigenetic and genetic) in combination with pharmacological modulators of ER stress/UPR, for enhancing anticancer efficacy while decreasing adverse effects. In conclusion, the identification of novel signals and mechanisms that are involved in the activation of the ER stress/UPR in acute leukemias will certainly help us both in understanding how neoplastic cells cope with those phenomena that provide them with a protective shield as well as in developing novel effective therapeutics. 

## Abbreviations

ABC	ATP-binding cassette
ABL1	Abelson 1
AEBSF	4-(2-Aminoethyl)benzenesulfonyl fluoride hydrochloride
AICAR	5-Aminoimidazole-4-carboxamide ribonucleotide
ALL	Acute lymphoblastic leukemia
AML	Acute myelogenous leukemia
AMPK	AMP-activated kinase
APL	Acute promyelocytic leukemia
ATF4	Activating transcription factor 4
ATF6α	Activating transcription factor 6α
ATO	Arsenic trioxide
BAD	Bcl-2 associated agonist of cell death
B-ALL	B-cell ALL
Bcl-2	B-cell lymphoma-2
BCR	Breakpoint cluster region
BIM	Bcl-2 interacting mediator of cell death
BiP	Binding immunoglobulin protein
BM	Bone marrow
bZiP	Basic leucine zipper domain
CAR	Chimeric antigen receptor
CK2	Casein kinase 2
CDK4	Cyclin-dependent kinase 4
C/EBPα	CCAAT/enhancer binding protein α
C/EBPε	CCAAT/enhancer binding protein ε
CXCL12	C-X-C motif chemokine 12
CHOP	C/EBP-homologous protein
2-DG	2-Deoxy-D-glucose
eIF2α	Eukaryotic translation initiator factor-2α
ER	Endoplasmic reticulum
ERK	Extracellular-regulated kinase
ERp57	ER protein of 57 kDa
ERAD	ER-associated degradation
ETV6	ETS-leukemia virus 6
FLT3	FMS-like tyrosine kinase 3
FLT3-ITD	FLT3- internal tandem duplication
GADD34	Growth arrest and DNA damage-inducible protein 34
GRP78	78-kDa glucose-regulated protein
HDAC3	histone deacetylase 3
HPCs	Hematopoietic progenitor cells
HSCs	Hematopoietic stem cells
IKZF1	Ikaros family zinc finger protein 1
IRE1α	Inositol-requiring enzyme-1α
JNK	c-Jun N-terminal kinase
LSCs	Leukemic stem cells
MAD1	Max dimerization protein 1
Mcl-1	Myeloid cell leukemia 1
MEK	Mitogen-activated protein kinase kinase
miR	MicroRNA
MLL	Mixed lineage leukemia
MM	Multiple myeloma
MPO	Myeloperoxidase
mTOR	Mechanistic target of rapamycin
MSC	Mesenchymal stromal cell
NCOR1	Nuclear receptor corepressor 1
NEDD8	Neural precursor cell expressed, developmentally down-regulated 8
NF-kB	Nuclear factor-kB
NOTCH1	Neurogenic locus notch homolog protein 1
NOXA	Phorbol-12-myristate-13-acetate-induced protein 1
N-Ras	Neuroblastoma-Ras
NPM1	Nucleophosmin 1
OSGEP	O-sialoglycoprotein endopeptidase
PAX5	Paired box 5
Ph^+^	Philadelphia-positive
Ph^-^	Philadelphia-negative
PML-RARα	Promyelocytic leukemia-retinoic acid receptor α
PML-NBs	PML nuclear bodies
PUMA	p53 upregulated modulator of apoptosis
NFR2	Nuclear factor (erythroid-derived 2)-like 2
PERK	Protein kinase RNA-like endoplasmic reticulum kinase
RA	Retinoic acid
RARα	Retinoic acid receptor α
RIDD	Regulated IRE1α-dependent decay
RNase	Endoribonuclease
ROS	Reactive oxygen species
RUNX1	Runt-related transcription factor 1
STAT3	Signal Transducer and Activator of Transcription 3
Syk	Spleen tyrosine kinase
S1P	Site-1 protease
S2P	Site-2 protease
T-ALL	T-cell ALL
TKI	Tyrosine kinase inhibitor
UBC6	Ubiquitin-conjugating enzyme 6
UBCH8	Ubiquitin-conjugating human enzyme 8
UFD1	Ubiquitin fusion degradation 1
URP	Unfolded protein response
XBP1	X-box binding protein
